# Obstructive shock caused by infection of a mediastinal tumor: a case report and literature review

**DOI:** 10.3389/fmed.2026.1726710

**Published:** 2026-03-03

**Authors:** Wenxin Wang, Luping Cheng, Xia Hu, Hong Wan, Chuanliang Pan

**Affiliations:** Department of Intensive Care Unit, The Third People’s Hospital of Chengdu, Affiliated Hospital of Southwest Jiaotong University, Chengdu, China

**Keywords:** CT, infectious, mediastinal tumor, obstructive shock, PICCO

## Abstract

**Background:**

Mediastinal tumor are a category of neoplasms that arise in the central region of the thoracic cavity. The clinical manifestations of this condition are diverse and have the potential to precipitate a range of severe complications, including obstructive shock. A comprehensive understanding of the etiology of mediastinal tumor is imperative for the timely recognition and intervention.

**Case report:**

The present case study focuses on a 38-year-old female patient who exhibited symptoms of chest tightness and dyspnea, devoid of any discernible precipitating factors. A chest CT scan revealed a mediastinal mass, which the patient initially disregarded. As symptoms worsened, her dyspnea significantly intensified, prompting presentation to the Huaxi Emergency Department. The mediastinal tumor was assessed as unresectable and was compressing the heart and major vessels, exacerbating the patient’s symptoms. Preliminary diagnostic investigations confirmed the presence of obstructive shock, the etiology of which was infection of the mediastinal tumor. Following the administration of anti-infective therapy, the implementation of symptomatic management, and the undertaking of appropriate monitoring, X-ray and CT scans demonstrated a reduction in tumor size.

**Conclusion:**

Obstructive shock caused by mediastinal tumor infection is relatively rare. This case review demonstrates that clinical CT scans, continuous cardiac output monitoring via pulse-wave-derived cardiac output catheter (PICCO), and assessment of clinical laboratory indicators hold significant value for early diagnosis in patients.

## Introduction

The wide variety of mediastinal tumor presents significant challenges in clinical management due to their complexity and diversity. Particularly when these tumor induce infectious necrosis, which may lead to severe obstructive shock and pose a life-threatening risk to patients.

The pathogenesis of infectious necrosis is multifactorial in nature, involving the increased metabolic demands of tumor cells, hypoxia, and tumor-associated inflammatory responses (1). The collective action of these factors contributes to tumor tissue necrosis and infection, thereby initiating systemic inflammatory response syndrome (SIRS), which may precipitate obstructive shock. Such obstruction may result from pulmonary embolism, tension pneumothorax, cardiac tamponade, or direct tumor compression (2, 3).

The clinical presentation of obstructive shock resulting from infectious necrosis commonly manifests as acute respiratory distress, hypotension, and altered mental status (4). Its incidence is rare, and its management poses significant challenges. These strategies encompass the prompt execution of imaging investigations, the utilization of fine-needle aspiration biopsy for diagnostic confirmation, and the fostering of collaboration among multidisciplinary teams (5). In addition, the following elements are integral to managing the condition: antibiotic therapy targeting the infection, symptomatic supportive care, and surgical intervention when necessary (6).

## Case report

**FIGURE 1:**
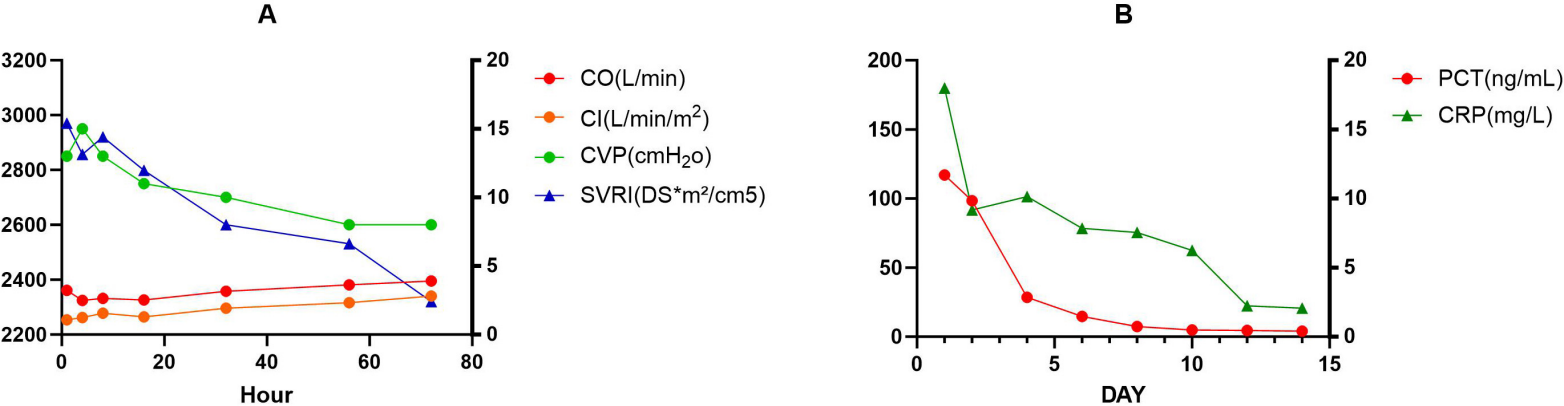
Two graphs labeled **(A,B)** compare different physiological parameters over time. **(A)** Shows four metrics: cardiac output (red), cardiac index (orange), central venous pressure (green), and systemic vascular resistance index (blue) over 80 h. Cardiac output and systemic vascular resistance index initially decrease, while central venous pressure remains stable. **(B)** Shows procalcitonin (red) and C-reactive protein (green) levels over 15 days. Both decrease significantly in the initial days, stabilizing afterward. Graph A and B have respective left and right Y-axes for measurement units.

**FIGURE 2:**
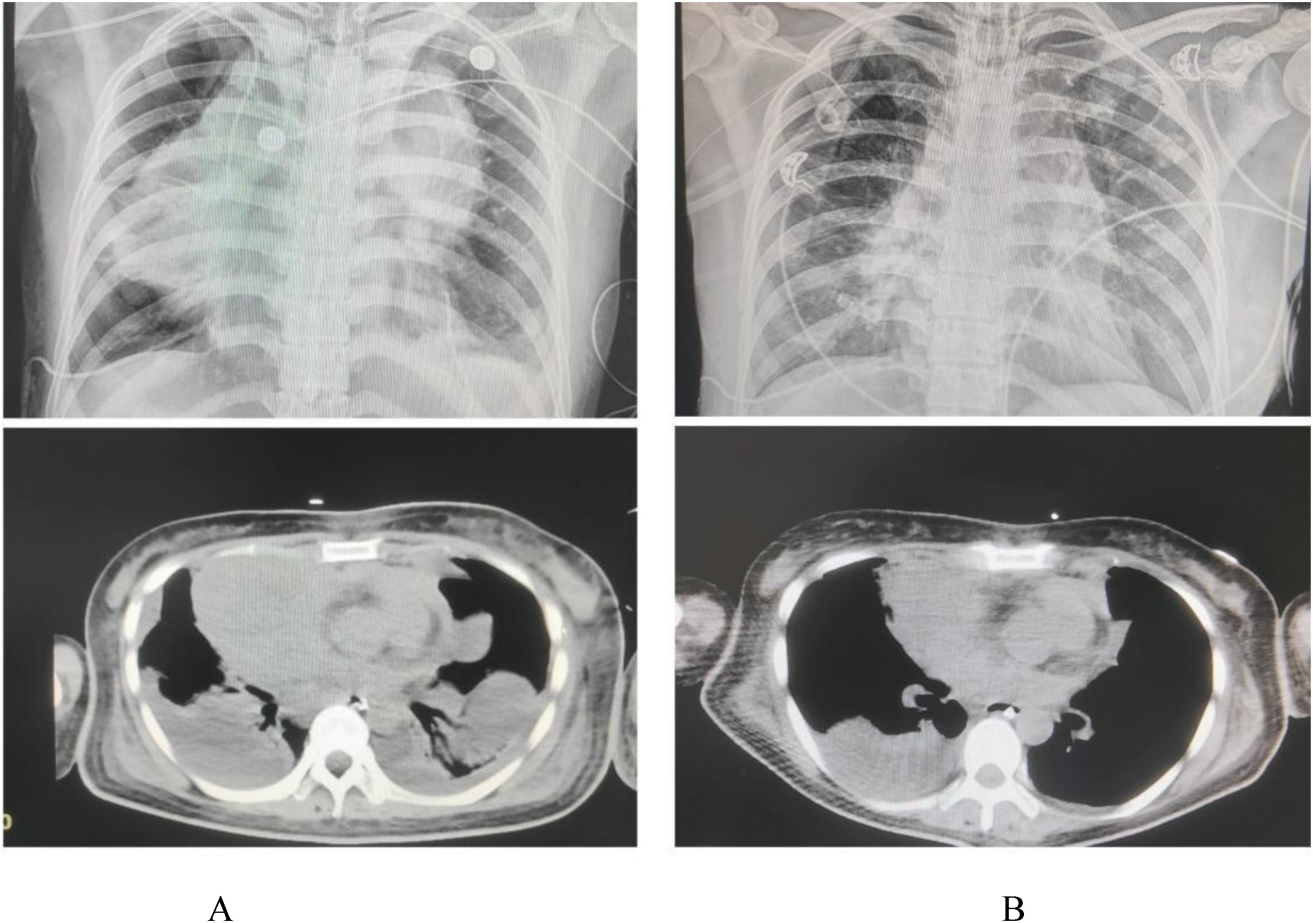
**(A)** Shows the patient’s initial CT scan and X-ray upon admission, while **(B)** displays the CT scan and X-ray taken 15 days later.

**FIGURE 3:**
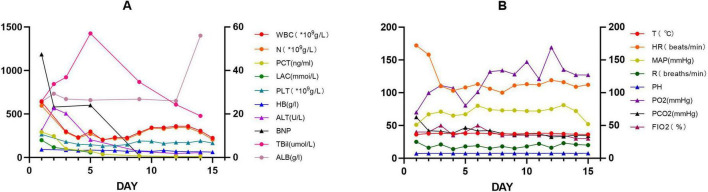
Two line graphs labeled **(A,B)** show various medical parameters over 15 days. **(A)** Tracks WBC, neutrophils, PCT, lactate, platelet count, hemoglobin, ALT, BNP, total bilirubin, and albumin. **(B)** Tracks temperature, heart rate, mean arterial pressure, respiratory rate, pH, PO_2_, PCO_2_, and FIO_2_. Each parameter is represented by different colored lines with data points indicating changes overtime.

**FIGURE 4:**
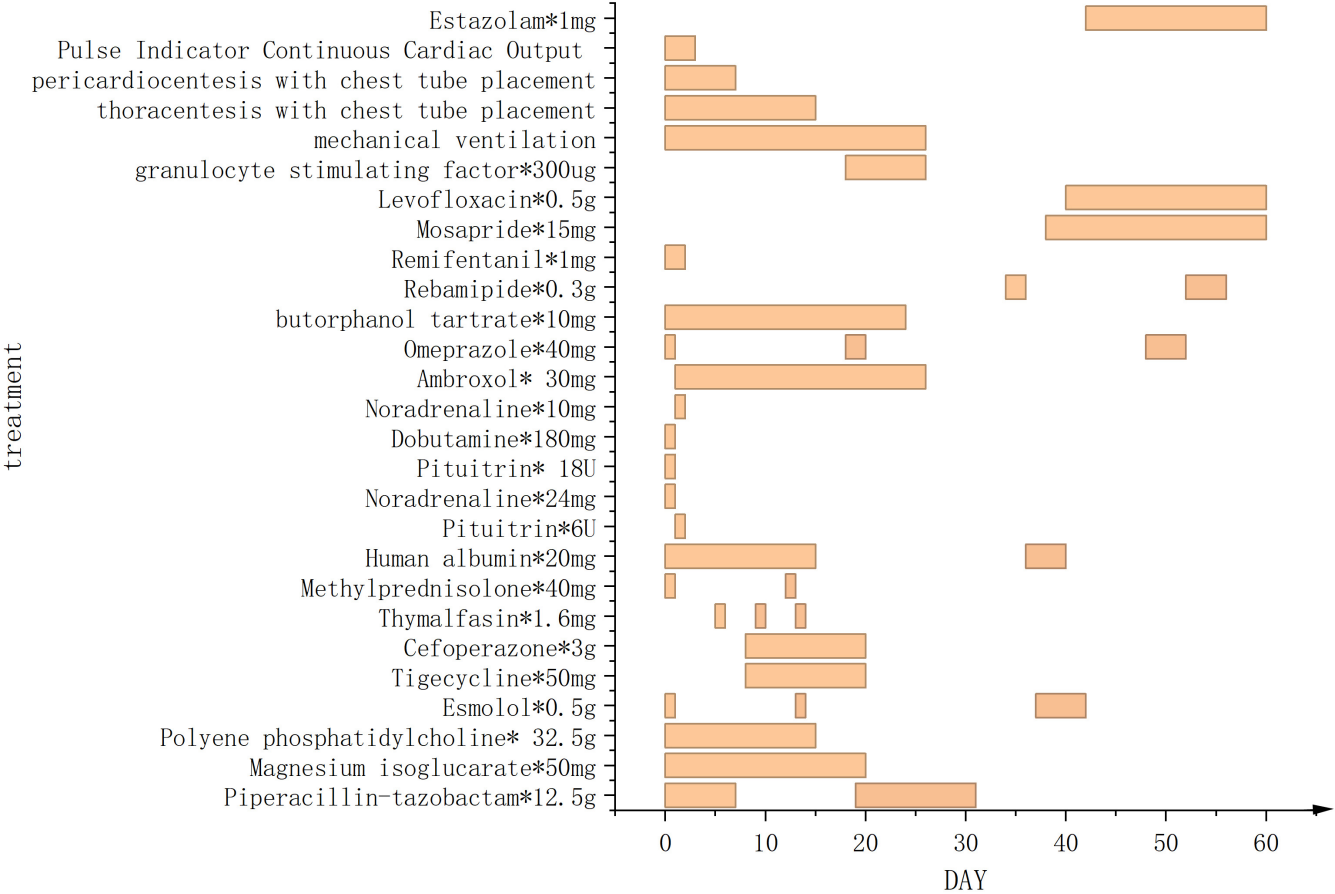
Treatment administered during the 60-day hospital stay (except for chemotherapy drugs).

This case report details a 38-year-old female patient who, on 1 July 2024, presented with chest tightness and shortness of breath without an apparent cause, accompanied by an occasional dry cough. The patient reported no fever, productive cough, or hemoptysis. A chest CT scan revealed a mediastinal mass, which the patient did not regard as significant. On 20 October 2024, the patient presented to the Huaxi Emergency Department with markedly worsened dyspnea. Further CT investigation confirmed an unresectable mediastinal tumor. Compression of the right superior pulmonary artery, right superior pulmonary vein, left jugular vein, and cardiac structures resulted in tachypnea and diminished responsiveness to verbal stimuli, necessitating hospitalization. Physical examination: The patient’s temperature was recorded at 36.2 °C. Their pulse rate was 172 beats per minute, and their respiratory rate was. The patient was breathing at a rate of 36 times per minute, and their blood pressure was recorded at 62/45 mmHg. However, the patient’s oxygen saturation levels were not measurable. The patient’s acute facial appearance is characterized by pupils that are bilaterally equal in size and round, with intact light reflexes. The patient was comatose and had a GCS score of 10. No anterior chest wall protrusion or retraction was identified. The patient’s heart rate was recorded at 172 beats per minute, indicating a regular rhythm. No murmurs were detected during cardiac auscultation. The presence of moist rales has been observed to result in a diminution of bilateral lung sounds. Throughout the entire process, low skin temperature was recorded, with no evidence of mottling. Following admission to the intensive care unit, the patient underwent several procedures, including endotracheal intubation with invasive mechanical ventilation, thoracentesis with chest tube placement, pericardiocentesis with chest tube placement, and arterial catheterization. Given the poor cardiac function identified by bedside ultrasound and the critical nature of the patient’s condition, continuous cardiac output monitoring via the Pulse Indicator Continuous Cardiac Output (PICCO) system was initiated with family consent. The invasive measurements comprised of blood pressure, cardiac function, and peripheral resistance indices [cardiac output (CO) 3.22 L/min, cardiac index (CI) 2.016 L/min/m^2^, systemic vascular resistance index (SVRI) 2970 DS*m^2^/cm^5^, central venous pressure (CVP) 13 cmH_2_O, invasive pressure monitoring 62/45 mmHg]. The maintenance of circulation was achieved through the administration of norepinephrine, pituitrin, and dobutamine, while esmolol was utilized to regulate the ventricular rate. The infection was treated with piperacillin-tazobactam, while acid suppression was managed with omeprazole. Ambroxol was administered for expectoration, while Polyene phosphatidylcholine and Magnesium isoglucarate were used to improve liver function, and human albumin infusion was given to alleviate pulmonary edema. The administration of vasoactive agents and esmolol was discontinued on the second day. Effective anti-infective therapy, guided by bacterial culture (Supplementary Figure 1) and symptomatic drug management, combined with monitoring of CO, CVP, CI, and SVRI over the first 3 days (Figure 1A), along with strict fluid balance management (Supplementary Figure 2), successfully eradicated the pathogen and halted further necrosis. On day 4, comprehensive chest CT and radiography (Figure 2A) revealed a 14.7 × 8.1 cm tumor. Subsequent pathological findings are shown in Supplementary Figure 3. The patient’s infection markers decreased: procalcitonin (PCT) fell from 12 to 0.41 ng/mL and C-reactive protein (CRP) from 180 to 20.79 (mg/L) (Figure 1B). Tissue edema resolved, and tumor necrosis was controlled. Follow-up CT and X-ray examinations 13 days later revealed tumor shrinkage to 10.7 × 6.3 cm (Figure 2B). Following 13 consecutive days of anti-infective therapy in conjunction with relevant symptomatic treatment, a reduction in the size of the patient’s mediastinal tumor was observed. The presence of infection was indicated by the presence of markers PCT and CRP, alongside imaging studies and subsequent pathological examination, which confirmed the presence of infectious necrosis within the mediastinal tumor. The patient was temporarily able to achieve remission and commenced cyclical chemotherapy under the guidance of an oncologist on day 14, with multidisciplinary support. In light of the patient’s critical condition and with the informed consent of the family, the oncologist opted for a combination of chemotherapy and immunotherapy (Ifosfamide Ifosphamide 2 g on days 14, 15, and 16; Mucosolvan 1.2 g on days 14, 15, and 16; Epirubicin 70 mg on day 14; Tislelizumab 100 mg on days 14 and 15). Considering the tumor remained unconfirmed, a single cycle of chemotherapy was administered initially, with uncertain tumor response. The patient was successfully weaned off mechanical ventilation, despite the presence of a tumor measuring 10.7 × 6.3 cm. During the 2-month treatment period, the patient demonstrated a positive progression, transitioning from an initial comatose state at admission to achieving independence in performing certain activities of daily living without the need for mechanical support. After hospital discharge, the patient will undergo chemotherapy treatment, overseen by an oncologist. A comprehensive review of patient monitoring and treatment was conducted, covering laboratory test data during the first 15 days after admission (Figure 3A), vital signs and blood gas analysis (Figure 3B), as well as therapeutic management throughout the hospitalization period (Figure 4).

## Discussion

Mediastinal tumor are growths that develop within the mediastinum. Tumor growth frequently leads to tumor cell necrosis and infection. A complex interplay exists between tumor cell necrosis and infection. Simple tumor necrosis is typically the result of inadequate blood supply due to accelerated proliferation of tumor cells (7). tumor necrosis is characterized by the presence of cellular death within the tumor tissue, in conjunction with an inflammatory response, which may manifest as fever and elevated C-reactive protein (CRP) (8). In the case of infectious tumor necrosis, significant signs of infection may be present, including fever, marked leukocytosis, elevated C-reactive protein, and increased procalcitonin levels (9–11). Furthermore, imaging studies such as CT or MRI can assist in assessing tumor size, morphology, extent of necrosis, and signs of infection, thereby providing crucial diagnostic clues (10, 12). The integration of infection markers, imaging studies, and relevant fluid cultures (12, 13) has been demonstrated to enhance diagnostic accuracy.

Obstructive shock manifests as significant symptoms and signs resulting from hemodynamic impairment, including acute dyspnea, chest pain, altered mental status, and cold, clammy skin, stemming from inadequate tissue perfusion (2, 4). Furthermore, cardiac signs such as faint heart sounds and arrhythmia may indicate compromised cardiac function (14). It is the responsibility of clinicians to identify cases through meticulous history-taking and physical examination. Furthermore, the utilization of rapid ultrasound screening (RUSH) and alternative imaging modalities has been demonstrated to facilitate the confirmation of the underlying cause of shock, including pericardial effusion, pneumothorax, or major vascular obstruction (15). Obstructive shock is characterized by rapid hemodynamic deterioration; early recognition and management of underlying obstructive pathology can significantly impact morbidity and mortality (16, 17). The PICCO indicator, as a dynamic monitoring technique, provides real-time cardiac output and other hemodynamic parameters, thereby better reflecting the patient’s circulatory status and aiding in the identification of shock causes (18, 19). It enhances the diagnosis of obstructive shock and guides treatment.

In the early stages of infection and necrosis in mediastinal tumor, symptoms such as fever, cough, and sputum production may occur. Given the critical nature of the condition, it can lead to obstructive shock, presenting with altered mental status, cold and clammy skin, and other signs of inadequate perfusion. The diagnosis of such cases is often facilitated by a combination of infection markers, including C-reactive protein and procalcitonin, tissue fluid cultures, and CT imaging findings, in conjunction with abnormalities detected through PICCO monitoring. During the acute phase when surgery is contraindicated, initiate antimicrobial therapy, perform appropriate drainage procedures, and strictly manage fluid balance via PICCO monitoring. After shock correction, conduct comprehensive investigations to determine tumor characteristics, followed by chemotherapy, surgical intervention, or integrated treatment based on the patient’s condition.

Upon admission, the patient presented in shock with loss of consciousness. Despite multiple medical interventions (including thoracentesis, pericardiocentesis, and endotracheal intubation), the cause of shock remained unclear. With family consent, PICCO therapy was initiated due to cardiac dysfunction and shock. CT imaging revealed vascular encasement, leading to a preliminary diagnosis of obstructive shock. The patient’s elevated infection markers (PCT, CRP) in conjunction with imaging findings and relevant culture results suggested a probable infection associated with a mediastinal tumor. This mediastinal infection likely caused the obstructive shock. We administered antimicrobial and symptomatic treatment under strict monitoring, followed by a course of chemotherapy; However, it should be noted that treatment approaches for mediastinal tumor exhibit significant variability. The recommended management strategies for this condition include an initial biopsy to determine the nature of the tumor. Following this determination, an assessment of the requirements for surgical intervention or adjuvant chemoradiotherapy is recommended (20, 21). Treatment plans must be strictly tailored to the precise pathological characteristics of the patient’s tumor (15). This study has several limitations, including the absence of precise follow-up pathological data

## Conclusion

Obstructive shock caused by mediastinal tumor infection is relatively rare and presents with insidious clinical manifestations, necessitating early diagnosis and treatment. This case review demonstrates that clinical CT scans, continuous cardiac output monitoring via PICCO, and clinical laboratory assessments hold significant value for the early diagnosis of such patients.

## Data Availability

The original contributions presented in this study are included in this article/Supplementary material, further inquiries can be directed to the corresponding author.
