# Supplementary material for: Obstructive shock caused by infection of a mediastinal tumor: a case report and literature review
Source: Front Med (Lausanne). 2026 Mar 3;13:1726710. doi: 10.3389/fmed.2026.1726710 (PMC12993886; doi:10.3389/fmed.2026.1726710)
Supplement: Supplementary file 1 [file Data_Sheet_1.docx]

Figure s1


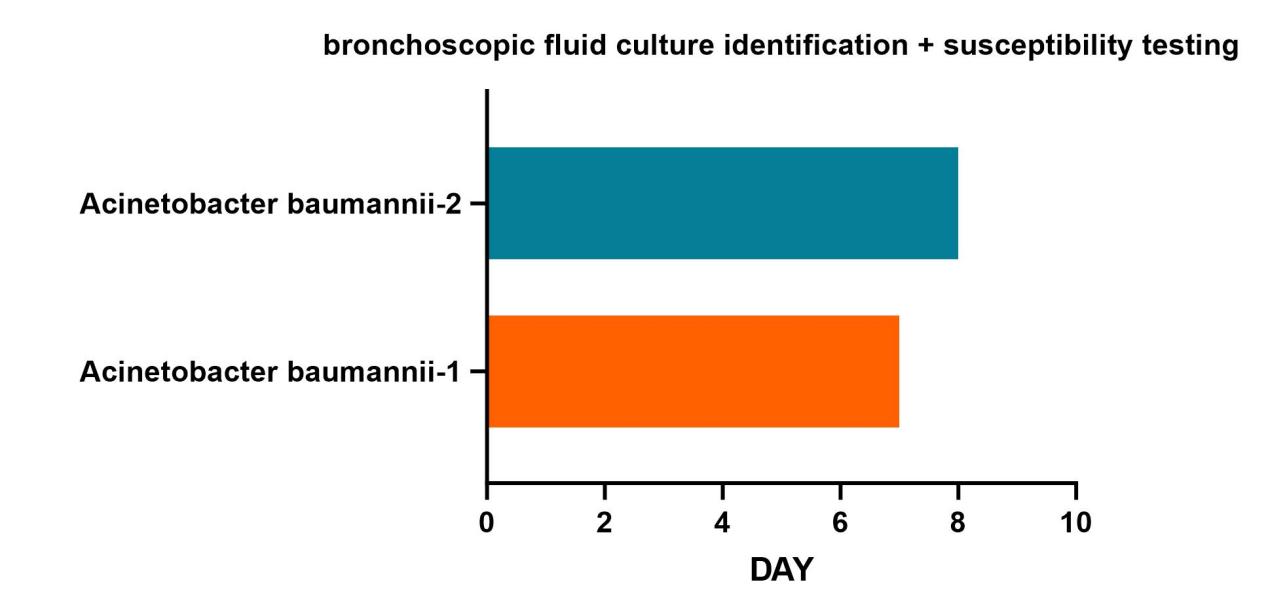


Acinetobacter baumannii-1 denotes treatment with piperacillin-tazobactam for infection from Monday 24th to 31st October 2024. Acinetobacter baumannii-2 denotes treatment with Cefoperazone and tigecycline for infection from 31st October to 7th November 2024.

Figure s2


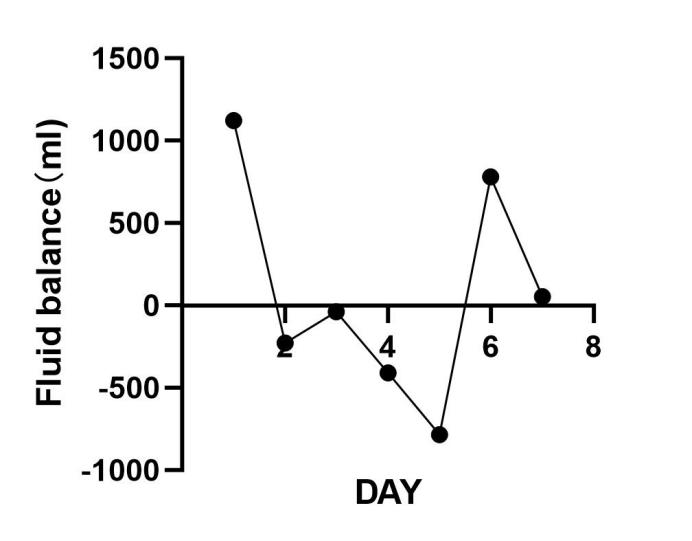


Fluid balance is calculated as the total fluid intake minus the total fluid output for the day.

Figure s3


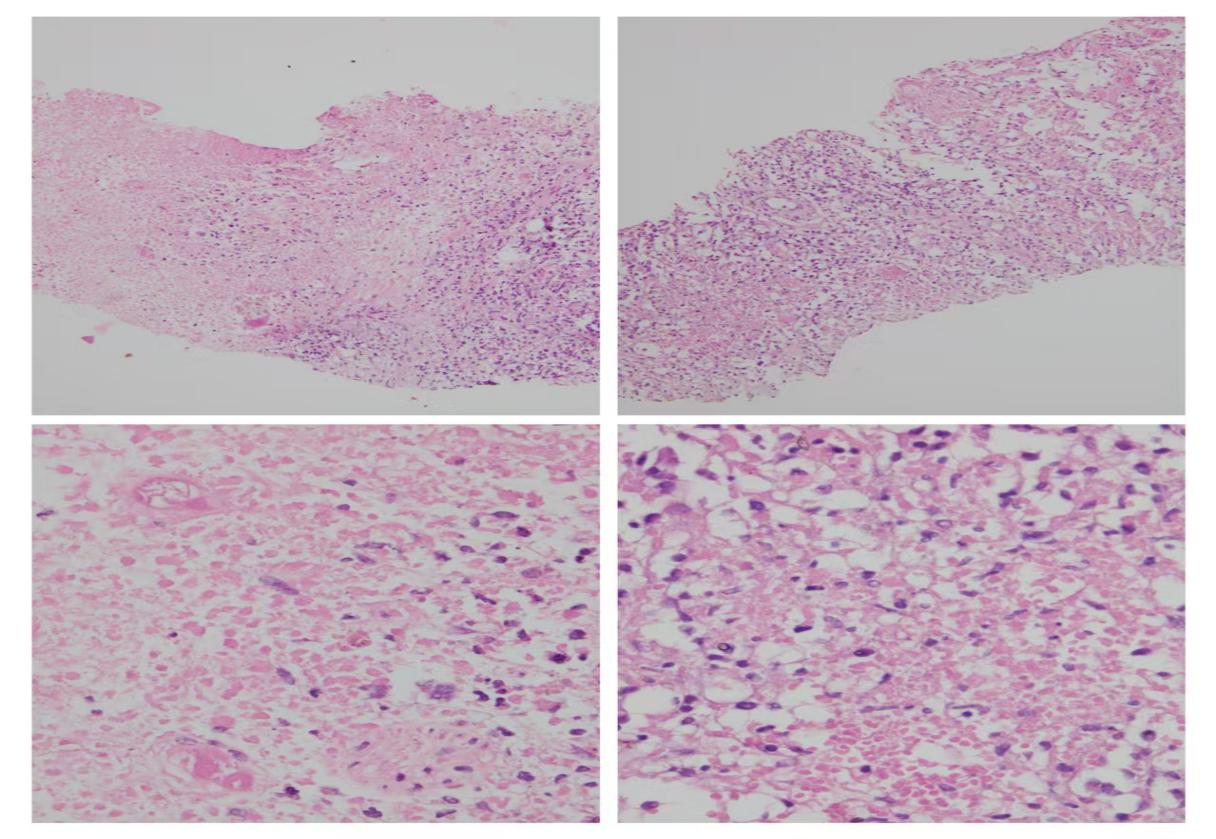


A B

Figure A shows the first pathological examination of the mediastinal tumour upon the patient's admission. Figure B shows the second pathological examination of the mediastinal tumour.Numerous necrotic areas contain histiocytes and lymphocytes. Immunohistochemistry supports a vascular origin, primarily suggesting pseudomyogenic hemangioendothelioma. Epithelioid hemangioendothelioma cannot be entirely excluded. Further genetic testing is recommended where necessary to confirm diagnosis(SERPINE1，FOSB, WWTR1-CAMTA1, YAP1-TFE3). Immunohistochemistry results: Des (–), CD34 (–), S100 (–), SMA (–), Ki-67 (+, 50%), CK7 (–), EMA (+), FLi-1 (+), STAT6 (partially +), ERG (+), CD21 (-), MDM2 (sporadically +), MyoD1 (-), CD31 (+), TFE3 (partially +), CKpan (-), FOSB (partially +).
